# Recombinant expression and characterisation of the oxygen-sensitive 2-enoate reductase from *Clostridium sporogenes*

**DOI:** 10.1099/mic.0.000568

**Published:** 2017-11-07

**Authors:** Pawel M. Mordaka, Stephen J. Hall, Nigel Minton, Gill Stephens

**Affiliations:** ^1^​ Bioprocess, Environmental and Chemical Technologies Research Group, Faculty of Engineering, University of Nottingham, University Park, Nottingham NG7 2RD, UK; ^2^​ Clostridia Research Group, BBSRC/EPSRC Synthetic Biology Research Centre (SBRC), School of Life Sciences, University of Nottingham, University Park, Nottingham NG7 2RD, UK; ^†^​ Present address: Centre for Synthetic Biology and Innovation, Department of Life Sciences, Imperial College London, South Kensington Campus, London SW7 2AZ, UK.

**Keywords:** 2-enoate reductase, 'ene'-reductase, bioreduction, *Clostridium sporogenes*, nitroalkene reduction, hydrogenation of carbon-carbon double bonds

## Abstract

‘Ene’-reductases have attracted significant attention for the preparation of chemical intermediates and biologically active products. To date, research has been focussed primarily on Old Yellow Enzyme-like proteins, due to their ease of handling, whereas 2-enoate reductases from clostridia have received much less attention, because of their oxygen sensitivity and a lack of suitable expression systems. A hypothetical 2-enoate reductase gene, *fldZ*, was identified in *Clostridium sporogenes* DSM 795. The encoded protein shares a high degree of homology to clostridial FMN- and FAD-dependent 2-enoate reductases, including the cinnamic acid reductase proposed to be involved in amino acid metabolism in proteolytic clostridia. The gene was cloned and overexpressed in *Escherichia coli.* Successful expression depended on the use of strictly anaerobic conditions for both growth and enzyme preparation, since FldZ was oxygen-sensitive. The enzyme reduced aromatic enoates, such as cinnamic acid or *p*-coumaric acid, but not short chain unsaturated aliphatic acids. The β,β-disubstituted nitroalkene, *(E)*-1-nitro-2-phenylpropene, was reduced to enantiopure *(R)*-1-nitro-2-phenylpropane with a yield of 90 %. By contrast, the *α,*β-disubstituted nitroalkene, *(E)*-2-nitro-1-phenylpropene, was reduced with a moderate yield of 56 % and poor enantioselectivity (16 % ee for *(S)*-2-nitro-1-phenylpropane). The availability of an expression system for this recombinant clostridial 2-enoate reductase will facilitate future characterisation of this unusual class of ‘ene’-reductases, and expand the biocatalytic toolbox available for enantioselective hydrogenation of carbon-carbon double bonds.

## Introduction

‘Ene’-reductases are being used increasingly in the pharmaceutical and agrochemical industries to produce chiral intermediates, pharmaceuticals and agrochemicals, since hydrogenation of carbon-carbon double bonds in activated, prochiral alkenes can introduce up to two new asymmetric centres in the product [1, 2]. The most widely used enzymes are flavoprotein oxidoreductases (‘Old Yellow Enzyme’-like proteins; OYEs), which accept α,β-unsaturated enals and enones as well as nitroalkenes [3]. Attempts have been made to obtain ‘ene’ reductases with broader substrate range and enantiocomplementarity by random and rational mutagenesis of classical OYE-like families [4]. Enzymes such as pentaerythritol tetranitrate reductase (PETNR) from *Enterobacter cloacae*, Old Yellow Enzyme-1 (OYE1) from *Saccharomyces pastorianus*, 12-oxophytodienoate reductase (OPR1) from *Lycopersicon esculentum* and nicotinamide-dependent cyclohexanone reductase (NCR) from *Zymomonas mobilis* have been engineered to alter their substrate ranges, improve activities and change stereoselectivities [4]. However, classical and engineered OYEs do not accept α,β-unsaturated free carboxylic groups or esters as substrates or these compounds are reduced poorly [3, 5]. Recently, a new method for synthesis of chiral α-substituted carboxylic acids from α-substituted α,β-unsaturated aldehydes was exemplified by employing biocatalytic hydrogen-borrowing enzyme cascades [3, 6]. However, development of such reactions is challenging, since both enzymes in the cascade must work concomitantly and under the same reaction conditions. Therefore, it would be desirable to identify enzymes that can reduce the enoates directly.

Relatively little attention has been paid to 2-enoate reductases from clostridia and other strict anaerobes [7, 8], because these enzymes are oxygen sensitive [9] and are considered difficult to work with [10]. However, 2-enoate reductases have broad substrate specificities and excellent stereospecificity for carbon-carbon double bond reduction within α,β-unsaturated carboxylates [11, 12]. Furthermore, a diverse range of 2-enoate reductases are available, since they are found in various proteolytic and non-proteolytic species such as *Clostridium kluyveri*, *Clostridium tyrobutyricum*, *Clostridium sporogenes*, and even in non-clostridial species, such as *Moorella thermoacetica* and *Peptostreptococcus anaerobius* [13–18]. The 2-enoate reductases from *C. tyrobutyricum* and *M. thermoacetica* show partial homology to a family of flavoproteins including enzymes such as 2,4-dienoyl-coenzyme A reductase from *E. coli* and OYEs from yeasts [18]. However, unlike classical OYEs, which have subunits with molecular weights of approximately 45 kDa, the 2-enoate reductases from *C. kluyveri*, *M. thermoacetica* and *C. tyrobutyricum* have subunit molecular weights of approximately 73 kDa. Furthermore, these enzymes contain flavin mononucleotide (FMN), like OYEs, but also possess an extra flavin adenine dinucleotide (FAD) and an iron-sulphur cluster [14, 18, 19].

Previously, we discovered that crude cell-free extracts of *C. sporogenes* DSM 795 catalyse highly enantioselective reduction of β,β-disubstituted nitroalkenes [20]. Thus, aryl derivatives were reduced in good yields (35–86 %), forming the respective *(R)*-nitroalkanes with almost perfect enantiomeric excess (ee). By contrast, α,β-disubstituted nitroalkenes were poor substrates, yielding the respective *(S)-*nitroalkanes in low yield (10–20 %) and with poor enantioselectivity (30–70 %). A new asymmetric methodology was proposed for preparation of γ-amino butyric acid (GABA) derivatives, which can be used to treat a range of central nervous system diseases [21]. The key step was the use of crude extracts of *C. sporogenes* for asymmetric reduction of *(Z)*-3-(4-chlorophenyl)-3-cyanopropenoic acid to *(S)*-3-(4-chlorophenyl)-3-cyanopropanoic acid, which was then used to synthesize the *(S)-*enantiomer of baclofen.

Future exploitation or engineering of clostridial 2-enoate reductases would depend on successful expression in a non-clostridial host, to avoid the need for strict anaerobiosis. Although a number of clostridial 2-enoate reductases [14, 15, 19] have been purified and characterised *in vitro*, none of the enzymes from proteolytic species has been successfully produced in *E. coli* by recombinant means. However, *E. coli* has been used to produce recombinant enzyme from *M. thermoacetica* under anaerobic conditions [18] and an 2-enoate reductase gene from *C. acetobutylicum* has been used in a heterologous metabolic pathway for production of hydrocinnamic acids aerobically, although the activity of the recombinant product was improved under anaerobic conditions [5]. Therefore, we identified the gene encoding 2-enoate reductase in *C. sporogenes* DSM 795, and demonstrated its successful expression in anaerobically grown *E. coli*. Using the recombinant protein produced, we demonstrated that this enzyme is indeed responsible for the enantioselective reduction of 2-enoates and nitroalkenes. The availability of a recombinant 2-enoate reductase will enable broader exploration of the potential industrial uses of this unusual enzyme.

## Methods

### Materials

Oligonucleotides and the synthetic DNA encoding *C. sporogenes* DSM 795 FldZ codon optimised for expression in *E. coli* were synthesised by Eurofins Genetic Service (Germany). Substrates and products of biotransformations were purchased from Sigma-Aldrich representing the highest purity grades available, with the exception of *(E)*-1-nitro-2-phenylpropene and *(E)*-2-nitro-1-phenylpropene and the corresponding racemic products, 1-nitro-2-phenylpropane and 2-nitro-1-phenylpropane, which were synthesised as described previously [20]. Media and media components were obtained from Melford (UK) and molecular biology reagents were from Qiagen (Germany) except where stated.

### Organisms and growth

*Clostridium sporogenes* DSM 795 was obtained from DSMZ (Deutsche Sammlung von Mikroorganismen und Zellkulturen GmbH, Germany). *E. coli* JM107 was purchased from Thermo Fisher Scientific (UK). *E. coli* BL21(DE3)pLysS competent cells were obtained from Merck Millipore (UK). *C. sporogenes* DSM 795 was cultivated in a medium containing cooked meat medium (5 g l^−1^; Lab M Limited), KH_2_PO_4_ (5 g l^−1^), l-cysteine HCl (0.5 g l^−1^), and resazurin (0.001 % w/v), with the addition of agar (20 g l^−1^) when required [20]. *E. coli* strains JM107 and BL21(DE3)pLysS were cultivated in LB agar or broth, adding 1 % glucose for BL21(DE3)pLysS only. Terrific Broth (TB) contained tryptone (12 g), yeast extract (24 g) and glycerol (4 ml) in 0.9 l of deionized water. The medium was autoclaved, cooled to room temperature and supplemented with phosphate buffer/glucose solution (100 ml, filter sterilised, containing glucose, 10 g, KH_2_PO_4_, 2.31 g and K_2_HPO_4_, 12.54 g). Anaerobic media were prepared and sterilized as described previously [22] and then transferred to a MACS anaerobic workstation (Don Whitley Scientific Limited), before inoculation, growth and storage.

### Genomic DNA extraction

An overnight culture of *C. sporogenes* (10 ml) was centrifuged at 3000 ***g*** for 10 min and the supernatant was discarded. The pellet was resuspended in PBS-lysozyme lysis buffer (180 µl; containing NaCl, 8 g l^−1^; KCl, 0.2 g l^−1^; Na_2_HPO_4_, 1.44 g l^−1^; KH_2_PO_4_, 0.24 g l^−1^; lysozyme, 10 g l^−1^) and incubated at 37 °C for 30 min, with occasional gentle agitation. RNase A solution (4 µl; 10 mg ml^−1^) was added and the sample was incubated at 37 °C for 15 min. Proteinase K solution (25 µl; >600 mAU ml^−1^), deionised water (85 µl) and sodium dodecyl sulphate solution (110 µl; 10 % w/v) were added. The sample was mixed by inversion and incubated at 65 °C for 30 min with occasional gentle agitation. Phenol:chloroform:isoamyl alcohol solution (25 : 24 : 1, saturated with 10 mM Tris, pH 8.0, 1 mM EDTA; Sigma) was added (400 µl) and mixed thoroughly by inversion. The sample was transferred to a phase-lock tube (phase-lock gel heavy 2.0 ml, Eppendorf), mixed, and centrifuged at 12 000 ***g*** for 3 min. The top layer was transferred into a fresh phase-lock tube and the phenol:chloroform:isoamyl alcohol extraction was repeated twice more. The top layer was transferred into a 1.5 ml microcentrifuge tube containing 3 M sodium acetate solution pH 5.2 (40 µl) and ice cold absolute ethanol (800 µl). The mixture was mixed gently but thoroughly and incubated at −80 °C for 30 min. The sample was centrifuged at 12 000 ***g*** for 15 min and the supernatant was poured off quickly to avoid resuspension of the pellet. The sample was washed by adding ethanol solution (1 ml; 70 % v/v) and centrifuged at 12 000 ***g*** for 3 min. The supernatant was poured off quickly and the DNA pellet was air dried for 45 min at room temperature. The pellet was dissolved in 50 µl of EB buffer (Qiagen).

### Cloning of *fldZ* reductase gene

Cloning was performed using standard molecular biology techniques. PCR was performed with FailSafe PCR System (Epicentre) using the *C. sporogenes* genomic DNA as a template with primers PM003 (5′-GGATGTACTACAAGTGCTATTCAC-3′) and PM008 (5′-AGTGCTGGATTTAGTCTAC-3′). The PCR product was cloned into pJET1.2 (Thermo Fisher Scientific) and sequenced. For protein overexpression, the *fldZ* gene was codon optimised for expression in *E. coli* with GENEius software (Eurofins MWG Operon) and synthesised with *Nde*I (at the 5′-end) and *Not*I (at the 3’-end) restriction sites. After restriction digestion with *Nde*I and *Not*I (Thermo Fisher Scientic) the insert from the cloning vector was ligated into pET20b(+) cut with the same restriction enzymes, to form the plasmid pET20b(+):*Cs*fldZ. Cloning of other expression plasmids is described in the Supplementary information 3.

### Protein overexpression in *E. coli* under anaerobic conditions

Expression of *fldZ* was analysed in anaerobic cultures of *E. coli* BL21(DE3)pLysS. The strain carrying pET20b(+):*Cs*fldZ was grown aerobically in LB supplemented with carbenicillin (50 µg ml^−1^) and chloramphenicol (34 µg ml^−1^). The culture was stored at −80 °C after adding 0.2 g glycerol per ml of culture. Pre-cultures were inoculated directly from the cryostock and were grown aerobically overnight in LB (10 ml) with the antibiotics and 1 % glucose at 37 °C with shaking at 200 r.p.m. Aliquots of the pre-cultures (100–500 µl) were used to inoculate the same medium (20 ml) in a sterile 50 ml plastic centrifuge tube and the cultures were incubated at 37 °C for about 6–7 h with shaking at 200 r.p.m. until the OD_600_ reached 0.9–1 (UVmini 1240 spectrophotometer, Shimadzu) and were kept at 4 °C overnight. The following day, the cells were collected by centrifuging at 3000 ***g*** for 10 min at 4 °C. The supernatant was discarded and the pellet was transferred into the anaerobic workstation. The pellets were used to inoculate anaerobic TB medium (100 ml) supplemented with antibiotics, glucose (final concentration 10 g l^−1^) and riboflavin (1 µg ml^−1^, filter sterilized) in a Duran screw cap bottle (100 ml) containing a magnetic flea. Cultures were mixed using a magnetic stirrer at 100 r.p.m. at 30 °C. When the OD_600_ reached 0.4–0.5, expression of proteins was induced by adding filter sterilized 1 M isopropyl β-D-1-thiogalactopyranoside (40 µl). Samples (2 ml) were taken before induction and at intervals after induction in order to determine the time point giving the highest protein yield. The cells were harvested by centrifugation at 3000 ***g*** for 10 min at 4 °C in the anaerobic workstation. Pellets were resuspended in anaerobic lysis buffer (250 µl ml^−1^ of the culture). The lysis buffer contained flavin adenine dinucleotide (0.1 µmol), riboflavin-5-phosphate (0.1 µmol), Benzonase Nuclease (1 µl; ≥250 U; Sigma), 10× BugBuster Protein Extraction Reagent (1 ml; Merck) and complete MiniEDTA-free protease inhibitor (1 tablet; Roche) dissolved in 10 ml of 100 mM potassium phosphate buffer pH 7.0. The lysis buffer was deoxygenated by sparging with nitrogen for at least 1 h. The cell samples were incubated in the anaerobic lysis buffer at room temperature for 10–15 min with shaking at 250 r.p.m. and additional vortexing every 2–3 min, and then centrifuged at 18 000 ***g*** for 20 min at 4 °C in sealed tubes outside the workstation. The supernatants (soluble protein fractions) were transferred into fresh tubes in the workstation. Insoluble pellets were resuspended in the original volume of the anaerobic lysis buffer and represented the insoluble protein fractions. Both protein fractions were analysed using SDS-PAGE. Samples (20 µl) were loaded onto the gel (Any kD Mini-PROTEAN TGX Precast Protein Gels, Mini-Protean electrophoresis system, Bio-Rad). The gels were stained using EZBlue Gel Staining Reagent (Sigma). Protein concentrations were determined using the DC Protein Assay (Bio-Rad). His-Bind Purification Kit (Novagen) and Pierce Protein Refolding Kit (Fisher) were used according to the manufacturers' instructions.

Expression of *fldZ* was also scaled up to 1 l to prepare cell-free extracts for enzymatic assays and biotransformations by increasing the volumes of culture media and buffers. The protein extracts were prepared in the lysis buffer described above (final volume 7 ml) except that the BugBuster Protein Extraction Reagent was omitted and a cell disruptor at 40 kpsi (Constant Systems Ltd) was used to lyse the cells. The protein extracts (about 5–6 ml) were collected under a flow of nitrogen gas in a 30 ml universal bottle. The samples were sealed immediately and transferred to the anaerobic workstation, poured to 50 ml plastic centrifuge tubes, sealed and centrifuged at 18 000 ***g*** for 20 min outside the workstation. The tubes were then transferred back to the workstation and the supernatants were collected for use in enzyme assays. For long term storage, FldZ was drop frozen and stored in liquid nitrogen.

### Enoate reductase assay

2-Enoate reductase activity of *E. coli* protein extracts was determined in a spectrophotometric assay, using anaerobic buffers and solutions that had been sparged with nitrogen for at least 1 h. The reactions were set up in the anaerobic workstation in gas-tight quartz cuvettes with silicone septa (Hellma-Analytics) containing protein extracts (20 µl), NADH (0.3 µmoles) in 50 mM potassium phosphate buffer pH 7.0 (880 µl). The cuvettes were pre-incubated at the desired temperature for 5 min in a temperature controlled spectrophotometer (Agilent 8453) and the reactions were started by injection of an anaerobic solution of substrate (1 µmole) in the same buffer or tetrahydrofuran (100 µl), as described in the text, using a gas-tight syringe. The initial rate of NADH oxidation was determined by measuring the decrease in absorbance at 340 nm using an extinction coefficient of 6220 M^−1^ cm^−1^.

### Biotransformations

Reduction of cinnamic acid and nitroalkenes was determined under anaerobic conditions in the workstation. All buffers and solutions were made anaerobic by sparging with nitrogen for at least 1 h prior to transfer into the workstation. Cinnamic acid reduction was measured by mixing the substrate (30 µmoles) dissolved in 50 mM potassium phosphate buffer pH 7.0 (6 ml), cell-free extract (20 mg protein) and NADH (36 µmoles) in 30 ml screw top vials sealed with silicone-PTFE caps. Reactions were shaken using an orbital shaking platform (200 r.p.m.; Grant Bio POS-300) at 40 °C. After times stated in the text, samples (2 ml) were taken, and centrifuged (13 000 ***g***, 1 min) outside the workstation. The supernatants were transferred into fresh 15 ml polypropylene centrifuge tubes (Falcon), acidified with 5 M HCl (0.2 ml) and extracted with diethyl ether (2.5 ml) containing an internal standard (*tert*-butylbenzene, 33 µmol). After centrifugation (3000 ***g***, 15 min) the water-saturated organic phase was dried by passing it through a 1 ml pipette tip containing anhydrous MgSO_4_. The dried sample (0.5 ml) was derivatised by adding methanol (0.1 ml), followed by a few drops (approx. 30 µl) of (trimethylsilyl)diazomethane solution (2.0 M in diethyl ether), until the mixture had a persistent yellow colour, and incubated for 30 min at room temperature. Acetic acid (15 µl) was added to quench the reaction. Samples were analysed by GCMS.

Nitroalkene biotransformations were performed in 30 ml screw top vials with silicone-PTFE caps. Each reaction contained the substrate (8 µmoles) in anaerobic *iso*-octane (4.8 ml) mixed with anaerobic 50 mM potassium phosphate buffer pH 7.0 (7.2 ml) containing protein crude extracts (20 mg), NADH (15 µmoles) and *tert*-butylbenzene (25 µl) as an internal standard. Reactions were shaken in the anaerobic cabinet at 200 r.p.m. at 40 °C for 24–72 h. Samples were taken from the organic phase, which separated readily by allowing to stand. Samples were analysed by liquid chromatography.

### Analytical methods

Cinnamic acid and phenylpropanoic acid samples were analysed by GC-MS (Agilent 7890A) equipped with a HP5-MS capillary column (0.25 mm by 30 m by 1 µm; Agilent) and an inert mass selective detector (Agilent 5975C) with a quadrupole mass analyser. Ultra-pure helium was used as carrier gas at 1.2 ml min^−1^ flowrate. Samples (1 µl, split ratio 100 : 1) were injected at 300 °C. The initial oven temperature was held at 45 °C for 5 min, then increased at 20 °C min^−1^ to 300 °C, and held for 10 min. Retention times of the internal standard *tert*-butylbenzene, substrates and products were compared to the retention times of authentic standards. Concentrations were calculated from calibration curves.

Samples of nitroalkenes (15 µl) and their reduction products in the *iso*-octane phase were analysed by HPLC (Agilent) using a Chiralcel OJ column (4.6 mm×250 mm ID) and hexane:isopropyl alcohol (9 : 1) as a mobile phase at 1 ml min^−1^ flowrate. Sample components were detected at 254 nm. Substrates and products were identified and quantified by comparing the retention times with authentic standards of racemic nitroalkanes, relative to the internal standard, *tert*-butylbenzene.

### GenBank accession numbers

The partial sequence of *C. sporogenes* genome containing *fldZ* was deposited in the GenBank as KT897281. The FldZ reductase amino acid sequence was deposited as AMQ78444. The sequence of plasmid pET20b(+):*Cs*fldZ containing the *C. sporogenes fldZ* gene codon optimised for expression in *E. coli* was deposited as KY369208.

## Results

### Identification of 2-enoate reductase in *C. sporogenes* DSM 795

**Fig. 1.**
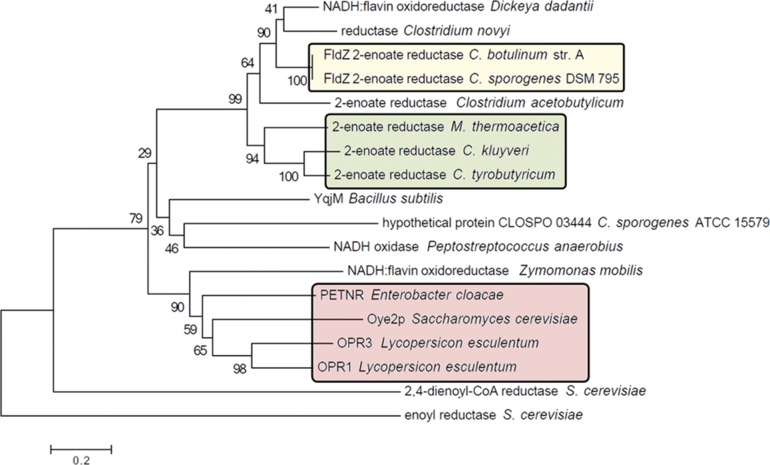
Evolutionary relationships of various carbon-carbon double bond reductases. The evolutionary history was inferred using the Neighbour-Joining method. The percentage of replicate trees in which the associated taxa clustered together in the bootstrap test (1000 replicates) is shown next to the branches. The tree is drawn to scale, with branch lengths in the same units as those of the evolutionary distances used to infer the phylogenetic tree. The evolutionary distances were computed using the Poisson correction method and are in the units of the number of amino acid substitutions per site. Evolutionary analyses were conducted in mega6 [41]. Yellow: 2-enoate reductases from proteolytic clostridia; green: previously characterized 2-enoate reductases from non-proteolytic organisms; red: classical OYE-like reductases. Sequences used to generate the tree were: NADH:flavin oxidoreductase *Dickeya dadantii* (C6C4J2); reductase *Clostridium novyi* (WP_011722529); fldZ reductase *C. botulinum* str. A (WP_011986666); 2-enoate reductase *Clostridium acetobutylicum* (WP_010966642); 2-enoate reductase *M. thermoacetica* (CAA76082); 2-enoate reductase *C. kluyveri* (CAA76083); 2-enoate reductase *C. tyrobutyricum* (CAA71086); YqjM *Bacillus subtilis* (P54550); hypothetical protein CLOSPO_03444 *C. sporogenes* ATCC 15579 (EDU37275); NADH oxidase *Peptostreptococcus anaerobius* (WP_002842930); NADH:flavin oxidoreductase (NCR) *Zymomonas mobilis* (WP_012817614); PETNR *Enterobacter cloacae* (3P7Y_A); Oye2p *Saccharomyces cerevisiae* (EDN62417); OPR3 *Lycopersicon esculentum* (NP_001233873); OPR1 *Lycopersicon esculentum* (NP_001234781); 2,4-dienoyl-CoA reductase *Saccharomyces cerevisiae* (CAA55506) and enoyl reductase *Saccharomyces cerevisiae* (NP_010269).

*C. sporogenes* DSM 795 contains an NADH-dependent 2-enoate reductase that belongs to the family of clostridial 2-enoate reductases containing FAD and an iron-sulphur centre [14]. The enzyme was proposed to be responsible for reduction of cinnamic acid to 3-phenylpropionic acid in the Stickland reaction [11]. This unusual metabolic pathway allows proteolytic clostridia to obtain carbon and energy for growth by coupling together the oxidation and reduction of pairs of amino acids. Although crude cell-free extracts of DSM 795 catalyse the reduction of nitroolefins, *(E)*-1-nitro-2-phenylpropene and *(E)-*2-nitro-1-phenylpropene, it has not been possible to purify the enzyme directly from *C. sporogenes* [20]. However, the structural similarities between cinnamate and nitroolefins and the known broad substrate range of all types of carbon-carbon double bond reductases [7, 23] may suggest that both can be reduced by the same enzyme. Therefore, we used a bioinformatics approach to search for 2-enoate reductase homologues in *C. sporogenes*. The genome sequence of *C. sporogenes* DSM 795 was not available when this work was initiated, so the genomes of closely related proteolytic strains *Clostridium botulinum* strain Hall A ATCC 3502 [24, 25] and *C. sporogenes* ATCC 15579 were analysed for the presence of genes encoding clostridial 2-enoate reductases, based on the sequences of previously identified enzymes in *M. thermoacetica* and *C. tyrobutyricum* [18]. One open reading frame in each genome, the *fldZ* gene in *C. botulinum* and CLOSPO_02780 in *C. sporogenes* ATCC 15579, showed significant homology to the *M. thermoacetica* and *C. tyrobutyricum* genes. PCR primers were designed to highly conserved regions located upstream and downstream of the consensus sequences of both genes (primers PM003 and PM008). These primers were used in PCR on *C. sporogenes* DSM 795 genomic DNA. The reactions yielded a 3.3 kb product of the expected size which was cloned into the pJET1.2 vector and sequenced. The sequence (Supplementary information 1; deposited in GenBank as KT897281) contained an open reading frame encoding 665 amino acids which shared 98 % identity and 99 % similarity to CLOSPO_02780 from *C. sporogenes* ATCC 1559, and 99 % identity and 99 % similarity to FldZ from *C. botulinum* str. Hall A ATCC 3502. The molecular weight of the enzyme subunit and its predicted amino acid composition were identical to enzymes purified from *C. kluyveri* and *C. sporogenes* and annotated as 2-butenoate and cinnamate reductases, respectively [11, 14, 15]. The predicted amino acid sequence of the DSM 795 FldZ showed that the N-terminal domain is similar to the classical OYE-like carbon-carbon double bond reductases (conserved NemA domain) and forms a FMN binding motif and substrate/catalytic site (Fig. S1, available in the online version of this article). Clostridial 2-enoate reductases also contain four conserved cysteine residues responsible for interaction with the iron-sulphur (Fe-S) cluster. The C-terminal part of the DSM 795 FldZ, which does not share any homology with OYE-like reductases, contains a small NADH binding domain within a larger FAD binding domain, a characteristic feature of clostridial 2-enoate reductases. Phylogenetic analysis revealed that the DSM 795 FldZ clusters most closely with the FldZ of *C. botulinum* str. Hall A ATCC 3502 (Fig. 1; yellow box) and then with 2-enoate reductases from other species (*C. tyrobutyricum*, *C. kluyveri* and *M. thermoacetica*). OYE-like reductases (red box) are much smaller proteins containing 329–402 amino acids, and these enzymes cluster separately. This suggests that DSM 795 FldZ is a member of the clostridial 2-enoate reductase family, with close similarity to the known enzymes. Although it shares a similar domain with OYEs, the enzyme is much less closely related to these enzymes than the clostridial enzymes, since it is much larger and contains both iron sulphur and FAD binding sites, in addition to the FMN binding site.

### Heterologous expression of the DSM 795 *fldZ* gene in *E. coli*

The *C. sporogenes* 2-enoate reductase gene was overexpressed in *E. coli* BL21(DE3)pLysS using the IPTG-inducible pET vector system. It has been suggested that the previous unsuccessful attempt to express 2-enoate reductase from *C. tyrobutyricum* was due to the low GC content of the DNA [18]. Therefore, the *fldZ* gene was optimised for expression in *E. coli* using the GENEius sequence adaptation tool and the modified *fldZ* gene sequence was synthesised *de novo*. Initial expression of the reductase using the pET20b(+):*Cs*fldZ construct using aerobically grown *E. coli* resulted in expression of insoluble protein only. Activity assays with cinnamic acid as the substrate conducted under anaerobic conditions showed that the recombinant protein was inactive (data not shown). Therefore, we investigated the effect of *fldZ* expression in *E. coli* grown under strictly anaerobic conditions. This resulted in the formation of mixture of soluble and insoluble FldZ with the expected size, about 73 kDa (data not shown). Furthermore, cinnamate reductase activity could be detected, with a specific activity of 378±22 nmol min^−1^ mg^−1^. No activity was detected in a control strain harbouring the empty expression plasmid.

In an attempt to purify the reductase, additional constructs were generated encoding FldZ tagged with the N-terminal 6xHis-tag, T7-tag, GST-tag, Strep-tag and S-tag as well as FldZ tagged on the C-terminus with 6xHis-tag (Supplementary information 3, Fig. S2). However, only the reductase with the N-terminal His-tag was expressed as a soluble protein and showed detectable activity in the cinnamic acid reduction assay. The specific activity was only 174±8 nmol min^−1^ mg^−1^, 46 % of the activity compared to the untagged enzyme. Activity was lost when the enzyme was purified using Ni-NTA resin (Novagen), although the enzyme was almost completely pure judging by the SDS-PAGE analysis. Addition of cofactors such as FAD (10 µM) and FMN (10 µM), or reducing agents such as dithiothreitol (10 mM) or tris(hydroxypropyl)phosphine (1 mM) to the buffers did not result in recovery of enzymatic activity. Attempts to refold the reductase using Pierce Protein Refolding Kit under anaerobic conditions were also unsuccessful. Since protein extracts of *E. coli* BL21(DE3)pLysS expressing the empty plasmid did not show cinnamate reductase activity, the properties of FldZ were further characterised by testing the enzymatic activity of unpurified protein extracts of *E. coli* expressing the untagged protein.

### Optimisation of the DSM 795 FldZ reductase activity

**Fig. 2.**
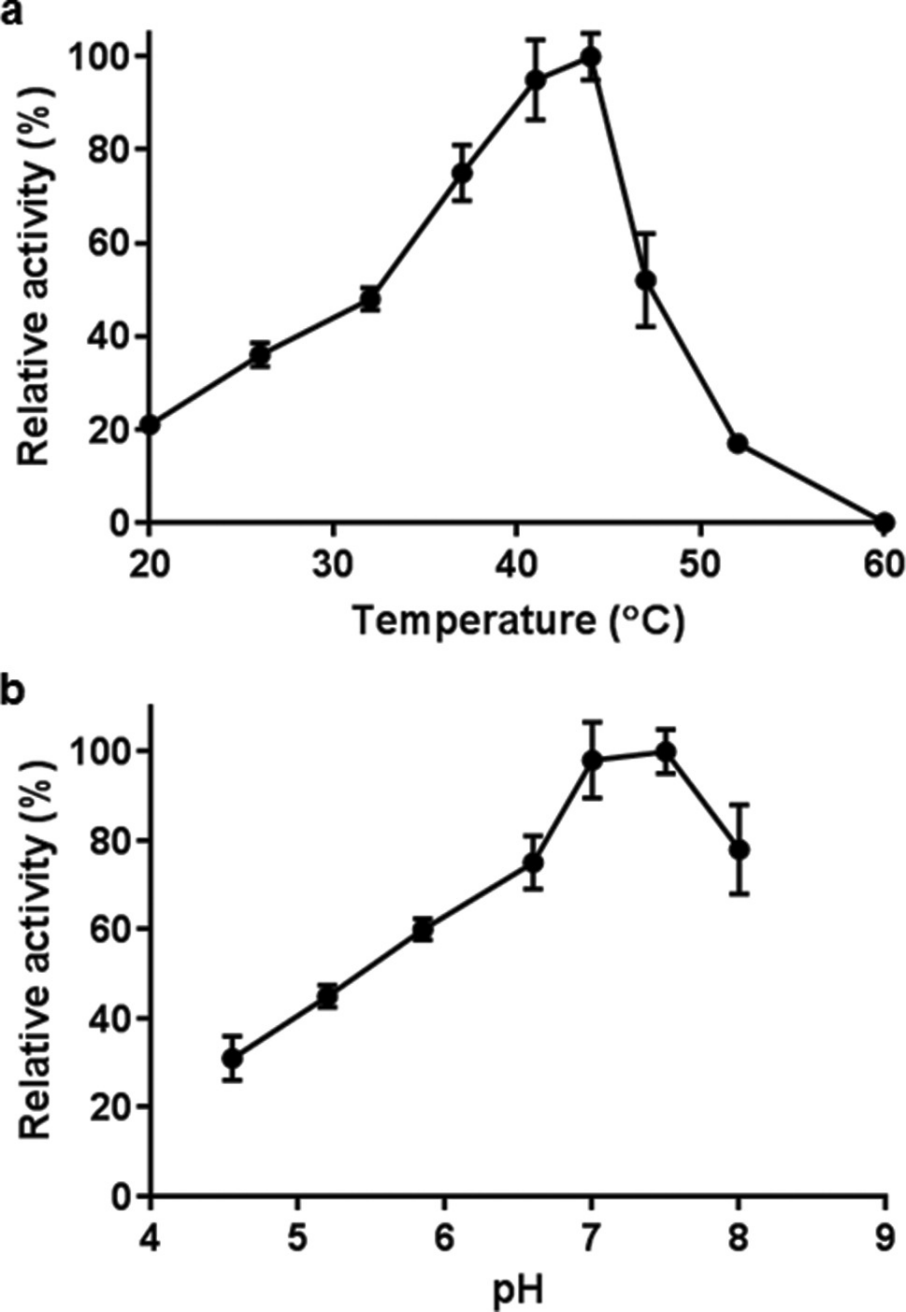
Temperature (a) and pH (b) profiles of FldZ activity in the 2-enoate reductase spectrophotometric assay. Cell-free extracts (20 mg) of *E. coli* expressing *fldZ* were used to oxidise NADH under anaerobic conditions in the presence of cinnamic acid. The reaction rate was determined from the decrease in absorbance at 340 nm. The effect of pH was tested in 50 mM potassium phosphate solutions adjusted to a range of pH values, with the lowest being outside the buffering range of 5.8–8.0. Error bars represent standard deviations of three independent experiments.

**Fig. 3.**
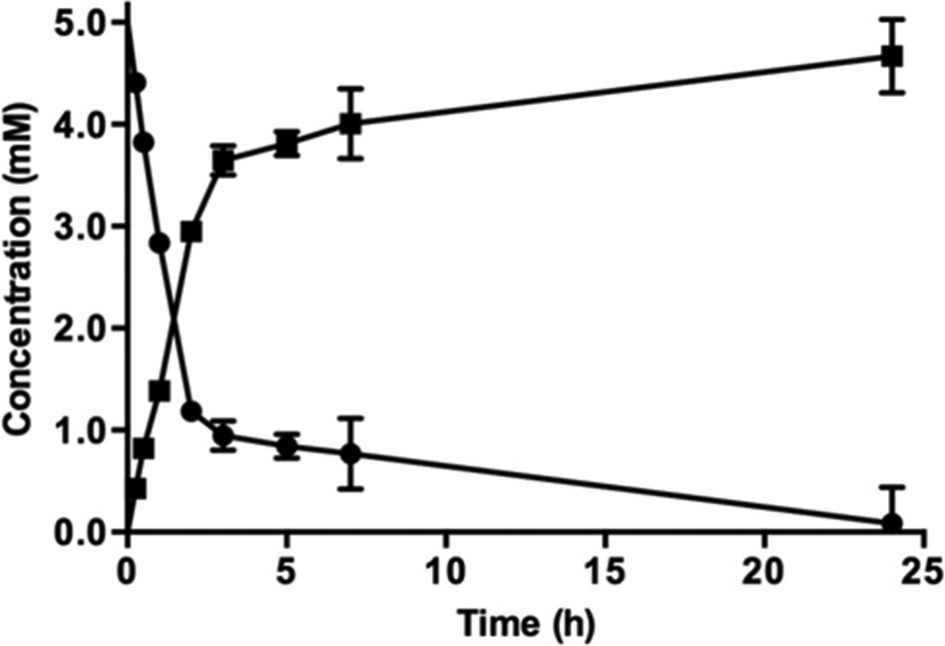
Anaerobic biotransformations of cinnamic acid using cell-free protein extracts of *E. coli* expressing *fldZ*. Reaction mixtures in 50 mM phosphate buffer (6 ml) contained cinnamic acid (30 µmoles) and NADH (36 µmol) as the electron donor. Error bars represent standard deviations of three independent experiments. Conversion of cinnamic acid (⚫) and formation of 3-phenylpropionic acid (■) were determined by derivatization to methyl esters and GC-MS.

**Fig. 4.**
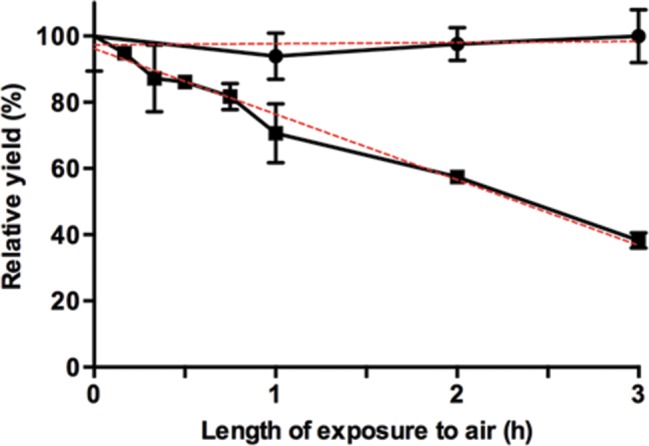
Inactivation of FldZ by air. Anaerobic biotransformations of cinnamic acid using cell-free protein extracts of *E. coli* expressing *fldZ*. Reaction mixtures in 50 mM phosphate buffer (6 ml) contained cinnamic acid (30 µmoles) and NADH (36 µmoles) as the electron donor. Error bars represent standard deviations of three independent experiments. Protein cell-free extracts were exposed to air, then degassed by sparging with nitrogen and used for reduction of cinnamic acid under anaerobic conditions. The yields of the biotransformations were normalized to the yield of the control sample, FldZ not exposed to air. Protein extracts exposed (■) and not exposed (⚫) to air.

Before investigating biotransformations, the conditions for optimal activity of FldZ were determined using an NADH-dependent spectrophotometric assay using cinnamic acid as the substrate. The highest reaction rate was observed at 40–45 °C (Fig. 2a). Higher temperatures caused a gradual decrease in FldZ activity, and the enzyme was completely inactive at 60 °C. The optimum pH for activity was between 7.0–7.5 (Fig. 2b). Therefore, further experiments were undertaken using 50 mM phosphate buffer pH 7.0 at 40 °C. The reduction of cinnamic acid to 3-phenylpropionic acid was measured directly by GC-MS, using NADH as the hydrogen donor, under anaerobic conditions. Cinnamic acid was reduced to 3-phenylpropionic acid at an initial rate of 1.35 µmol min^−1^ mg^−1^ (Fig. 3). The rate decreased after 2 h, but continued slowly until the yield of 3-phenylpropionic acid was almost 100 % after 24 h. No product formation was observed in control experiments with *E. coli* expressing empty pET20b(+). The effect of oxygen on FldZ activity was investigated by exposing samples of protein extracts to air for time intervals between 15 min and 3 h, keeping the samples on ice throughout the exposure time. The protein extracts were then tested for cinnamic acid reduction using NADH as the electron donor under anaerobic conditions at 40 °C, measuring substrate and product concentrations after 2 h by GC-MS (Fig. 4). FldZ lost 57 % of the activity after 2 h of exposure to air, and activity could not be restored by reintroduction of anaerobic conditions. When the enzyme was incubated under anaerobic conditions at 4 °C, there was no loss of activity, indicating that the DSM 795 FldZ enzyme was stable when stored on ice. Therefore, the enzyme is inactivated by exposure to air, although at a relatively slow rate.

### Substrate range of the DSM 795 FldZ

FldZ was tested for oxidation of NADH in the presence of unsaturated aromatic and aliphatic carboxylic acids using the spectrophotometric assay. Reactions containing protein extracts of *E. coli* expressing the empty pET20b(+) were used as controls to subtract the background activity of native *E. coli* oxidoreductases. Because of poor water solubility of some chemicals tested, stock solutions of all the substrates were prepared in tetrahydrofuran (THF). The rate of cinnamate-dependent NADH oxidation was similar in the presence and absence of 10 % THF, indicating that THF did not inhibit FldZ. The enzyme showed a narrow substrate range for reduction of 2-enoates. The highest rate of NADH oxidation was observed in the presence of cinnamic acid (Table 1). The presence of methyl groups in the alpha or beta positions drastically decreased the activity to about 1 %. *p*-Coumaric acid was a relatively good substrate (43 % activity when compared to cinnamic acid), but there was almost no activity when the aromatic ring was further substituted (caffeic acid and indoleacrylic acid). FldZ did not accept aliphatic enoates at all, suggesting that the aromatic ring plays a crucial role in the interaction between the enzyme and the substrate.

**Table 1.** Substrate range of the DSM 795 FldZ 2-enoate reductase. Cell-free protein extracts of *E. coli* expressing *fldZ* were used to oxidise NADH in the presence of unsaturated carboxylic acids dissolved in THF The enzyme activity was determined by measuring decrease in the absorbance at 340 nm. Protein extracts of *E. coli* expressing the empty expression vector were used to determine and subtract the background activities for each substrate of native *E. coli* oxidoreductases. Errors represent standard deviations of three independent experiments. nd, not detected.

**Substrate**	**Structure**	**Activity (nmol min^−1^ mg^−1^)**	**Relative activity**
cinnamic acid	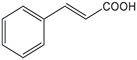	498±39	100 %
α-methyl-cinnamic acid	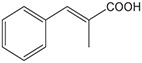	<3±0.1	<0.7 %
β-methyl-cinnamic acid	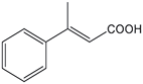	7.8±0.3	1.6 %
*p*-coumaric acid	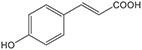	218.4±21	43.3 %
caffeic acid	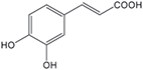	4.2±0.2	0.8 %
3-indoleacrylic acid	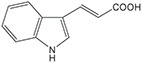	<2.4±0.3	<0.5 %
crotonic acid	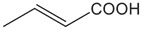	nd	–
2,3-dimethylacrylic acid	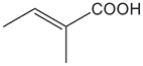	nd	–
3,3-dimethylacrylic acid	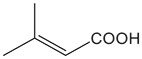	nd	–
2-methyl-2-pentenoic acid	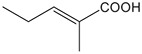	nd	–
sorbic acid	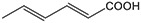	nd	–

Cell-free extracts of *E. coli* expressing *fldZ* were also used for reduction of *(E)-*1-nitro-2-phenylpropene and *(E)-*2-nitro-1-phenylpropene, in a biphasic biotransformation containing *iso*-octane to deliver the water-insoluble substrates. The samples were incubated at 40 °C and the yield and enantiomeric excess of reactions were determined by chiral HPLC (Table 2). Although extracts of the *E. coli* host containing the empty vector did not reduce cinnamic acid, there was detectable nitroalkene reductase activity. Thus, *E. coli* extracts containing the empty cloning vector reduced both (*E*)-1-nitro-2-phenylpropene and (*E)-*1-phenyl-2-nitropropene with poor yields (7 and 17 % after 72 h, respectively) and very modest enantiomeric excess (51 and 20 %). However, the expression of *fldZ* significantly improved biotransformations yields. FldZ showed >99 % enantioselectivity for reduction of (*E*)-1-nitro-2-phenylpropene, which was reduced in 90 % yield of *(R)*-1-nitro-2-phenylpropane. *(E)-*2-Nitro-1-phenylpropene was reduced to *(S)*-2-nitro-1-phenylpropane in 56 % yield, but with only 16–17 % ee. It is possible that the competing background nitroalkene reductase activity in the host may have contributed to the overall poor enantioselectivity. Therefore, further work is needed either to purify the enzyme by alternative strategies (e.g. conventional column chromatography) or to inactivate the interfering reductases in the expression host by one of many strategies available for generation of gene knock-outs in *E. coli*. Potential candidates include known *E. coli* reductases, such as NEM, which catalyses the reduction of unsaturated compounds, such as 1-nitrocyclohexene [26] or nitroreductases, which can also reduce carbon-carbon double bonds [27]. Nevertheless, there was no significant interference with the DSM 795 FldZ-catalysed reduction of (*E*)-1-nitro-2-phenylpropene, which was reduced with almost perfect enantioselectivity.

**Table 2.** Anaerobic reduction of phenylnitropropenes using DSM 795 FldZ reductase Biphasic reactions contained protein cell-free extracts of *E. coli* expressing *fldZ* and NADH (15 µmoles) in 50 mM potassium phosphate buffer (7.2 ml) and the substrate (8 µmoles) dissolved in *iso-*octane (4.8 ml). Control experiments contained cell-free protein extracts of *E. coli* transformed with the empty expression vector. Reaction yields and enantiomeric excess were determined by chiral HPLC. Errors represent standard errors of mean of three independent biotransformations.

**Substrate**	**Strain**	**Yield (%)**			**ee (%)**		
		**24** h	**48** h	**72** h	**24** h	**48** h	**72** h
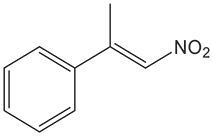 (*E*)−1-nitro-2-phenylpropene	*fldZ*	72.0±4.5	81.0±3.3	90.1±3.8	>99 *(R)*	>99 *(R)*	>99 *(R)*
	pET20b(+)	6.3±0.1	7.5±0.2	7.7±0.4	54.8±4.5 *(R)*	52.6±0.6 *(R)*	51.4±9.1 *(R)*
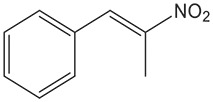 (*E)−*2-nitro-1-phenylpropene	*fldZ*	35.2±0.3	48.9±0.4	56.0±1.9	17.0±0.5 *(S)*	16.1±1.6 *(S)*	16.9±0.2 *(S)*
	pET20b(+)	10.2±4.2	17.2±2.8	17.0±0.6	16.8±5.5 *(S)*	17.2±1.6 *(S)*	20.3±2.3 *(S)*

## Discussion

Since the 1990s, carbon-carbon double bond reductases have attracted significant attention as biocatalysts for stereoselective hydrogenation of activated alkenes. Although the first reports on stereospecific reductions using *Clostridium* spp. were published in the 1970s [13], clostridial 2-enoate reductases have not been fully assessed for synthetically useful asymmetric hydrogenation. One of the key barriers is a widely held perception that it is difficult to grow and handle clostridia [10], despite their widespread use for biobutanol production at scale [28]. For this reason, we expressed the cloned DSM 795 *fldZ* gene in *E. coli*, to facilitate future exploitation of this unusual 2-enoate reductase.

2-Enoate reductase genes from the non-proteolytic strains *C. acetobutylicum* [5] and *M. thermoacetica* [18] have been expressed in *E. coli*, albeit without characterisation of either of the recombinant enzymes produced. Our report is the first example of successful production and isolation of an active recombinant 2-enoate reductase from a proteolytic *Clostridium* and its use for industrially relevant biotransformations. There are significant differences in codon usage between *E. coli* and clostridia [18], so expression studies were undertaken with a synthetic *fldZ* gene codon optimised for expression in *E. coli*. Although aerobic expression was unsuccessful, active FldZ could be isolated when the *E. coli* host was grown anaerobically. It should be noted that, unlike *C. sporogenes*, *E. coli* is a facultative anaerobe, so it may also be possible to grow the host aerobically and then switch to anaerobic conditions immediately before induction of *fldZ*. Similar approaches have been used previously to express oxygen sensitive systems in *E. coli* [29, 30]. When the aeration is stopped, anaerobic conditions develop rapidly, due to the rapid rate of oxygen consumption by actively growing cells, enabling expression of the active, oxygen sensitive system. In future work, this strategy can be investigated as a means to simplify FldZ production at scale.

The DSM 795 FldZ enzyme was stable *in vitro* under anaerobic conditions for at least 3 h without losing activity, but was irreversibly inhibited after exposure to air. This is a significant difference when compared with classical OYEs, which can be overexpressed under aerobic conditions and used for anaerobic biotransformations after deoxygenation [31]. Moreover, some OYEs are still active under aerobic conditions, although the presence of oxygen can change the enantiopurity of the reaction products [32]. The optimal temperature and pH of recombinant DSM 795 FldZ were similar to those reported previously using 2-enoate reductase isolated directly from *C. sporogenes* [11], but differ significantly from those reported for 2-enoate reductases from other anaerobes [14], showing a diversity in this class of enzymes.

Previously, 2-enoate reductases were purified directly from *Clostridium spp.* and other strains using a multi-step protein chromatography method [14, 18, 19], which was laborious and required specialised equipment for maintaining anaerobic conditions during the course of purification. To attempt easier purification, we decided to produce recombinant FldZ that incorporated affinity tags. In the past OYEs have been successfully overexpressed and purified as His-tagged proteins [33–35]. By contrast, our attempts to tag and purify FldZ using affinity chromatography techniques were unsuccessful. This may reflect the complexity of clostridial 2-enoate reductases, and their multiple cofactor content. Nevertheless, an inability to purify the enzyme would not prevent its exploitation in the future, since it is simpler to use whole cells expressing the enzyme, to solve problems with expensive cofactor recycling. However, the background activity of *E. coli* enzymes may interfere with stereospecificity and enantioselectivity of the reaction of interest. This should be carefully assessed for each substrate tested, and, if needed, the interfering reductases in the expression host can be inactivated.

The key advantage of using clostridial 2-enoate reductases is their ability to reduce less activated substrates containing carbon-carbon double bonds conjugated with carboxylic acid groups [13, 14, 18], which are often not reduced by classical OYEs [8]. We confirmed that the DSM 795 FldZ can reduce 2-enoates, although with a narrower substrate range than the enzymes from non-proteolytic clostridia and other genera. Thus, FldZ reduced cinnamic acid and *p*-coumaric acid at the highest rates, whereas α- and β-methyl cinnamic acid, caffeic acid and 3-indoleacrylic acid were extremely poor substrates, and aliphatic 2-enoates were not reduced at all. Further work is needed to test the scope to express other clostridial 2-enoate reductases in anaerobically-grown *E. coli*, to provide access to a broader range of alkanoates.

The DSM 795 FldZ enzyme was also used successfully to reduce prochiral nitroalkenes. The β,β-disubstituted nitroalkene, (*E*)-1-nitro-2-phenylpropene, was reduced to *(R)*-1-nitro-2-phenylpropane with high yields and excellent ee. The stereoselectivity of FldZ is similar to carbon-carbon double bond reductases such as yeast OYE1-3 [36], OPR1 from tomato [37], and KYE1 and YersER [35], but is opposite to PETNR [38], OPR3, YqjM [37] and NCR-reductase from *Zymomonas mobilis* [36]. The unpurified FldZ showed higher enantioselectivity than purified OYE1-3 from yeast and comparable enantioselectivity and activity to OPR1, KYE1 and YersER. By contrast, the *α,*β-disubstituted nitroalkene, (*E)-*2-nitro-1-phenylpropene, was reduced to *(S)-*2-nitro-1-phenylpropane with moderate yield and poor enantioselectivity. However, the yield of the product (56 %) was significantly improved when compared to the reduction using *C. sporogenes* DSM 795 protein extracts (yield 20 %) under similar reaction conditions [20].

*C. sporogenes* may contain enzymes other than FldZ that also catalyse nitroalkene reduction. Preliminary attempts to purify the nitroalkene reductase directly from *C. sporogenes* by conventional chromatography indicated the presence of more than one enzyme reducing *(E)*-2-nitro-1-phenylpropene (unpublished results). Furthermore, the analysis of genome sequences of *C. botulinum* ATCC 3502, *C. sporogenes* ATCC 15579 and recently published genomes of *C. sporogenes* NCIMB 10696 [39] and *C. sporogenes* DSM 795 [40] revealed the presence of an open reading frame showing features characteristic for OYE-like reductases (Fig. 1; CLOSPO_03444). This would be a perfect candidate for the second enzymatic activity, since homologous flavin containing reductases such as PETNR, KYE1, YersER and NRSal were found to reduce α,β-disubstituted nitroalkenes [27, 35, 38].

To conclude, we have shown that the *C. sporogenes* DSM 795 FldZ 2-enoate reductase has potential as an industrial biocatalyst due to its ability to catalyse the NADH-dependent reduction of a variety of activated alkenes in a stereoselective manner. It is an efficient catalyst for the reduction of aromatic 2-enoates as well as β,β- and *α,*β-disubstituted nitroalkenes.

## Supplementary Data

414 Supplementary File 1
