# Supplementary material for: Recombinant expression and characterisation of the oxygen-sensitive 2-enoate reductase from Clostridium sporogenes
Source: Microbiology (Reading). 2017 Nov 7;164(2):122–32. doi: 10.1099/mic.0.000568 (PMC5882074; doi:10.1099/mic.0.000568)
Supplement: Supplementary File 1 [file mic-164-122-s001.pdf]

SUPPLEMENTARY INFORMATION

Microbiology

Recombinant expression and characterisation of the oxygen-sensitive 2-enoate reductase from Clostridium sporogenes

Pawel Mordaka (1,3), Stephen J. Hall (1), Nigel Minton (2), Gill Stephens (1)

1. Bioprocess, Environmental and Chemical Technologies Research Group, Faculty of Engineering, University of Nottingham, University Park, Nottingham NG7 2RD, United Kingdom
2. Clostridia Research Group, BBSRC/EPSRC Synthetic Biology Research Centre (SBRC), School of Life Sciences, University of Nottingham, University Park, Nottingham NG7 2RD, United Kingdom
3. Present address: Centre for Synthetic Biology and Innovation, Department of Life Sciences, Imperial College London, South Kensington Campus, London SW7 2AZ United Kingdom

Corresponding authors:
Pawel Mordaka p.mordaka@imperial.ac.uk; Gill Stephens gill.stephens@nottingham.ac.uk

1. Identification of 2-enoate reductase in *C. sporogenes* DSM795

Sequence of *Cs*fldZ gene with flanking regions (3274 bp)

```
GGATGTACTACAAGTGCTATTCACTATTTTGAAAAAGAACATTTAATTGAAGTGGAAAAAGGAAAAAAATGGTCATCGTTATTA
TAATGTTGTAGATGTATTTCGATTGCTTTCTTATACAAAGTATCGTTCTATGGAAATACCTATGAAAACTATTATTGCACAAT
TTGGCGGGGAAGAAAATAATTATAAATTAATAGAAAAAAGAGAAACAATGTATCAATTAGAGGCCTTAAAAAGAGCCCAATAT
TATATGAATTTAGCAGATGCTATTGGGGAACATCTTGTTAGCATAAGAAGAATTGAAGAACTATTGAATAAGTATGAATTTGC
AAAGTCACCTGAAGTAACGATTATGTGTGATGATGAGTGTGGGTGGCTTTCAAAAAAGCGTAGTTCACAAAAGATAATTCATG
AATGGGTAAAAGCAATGCCAACAGTGCAGCTAGGGGTATTTGATTCAAGAATGGGAATGAGTAACTTTGGATATTTGGTAAAA
ACTAAAAAGCGAGAAGAATTAGAACTTCCGCTAGGGTTATATGCCAAAGAGCTAAAAAGTACATCTTGTATACATACAATTGT
AATGGCAGATGAAGATTTCACACAACAACCACAGAAGGTTTTTAAAAAGGCTTCTGAATTTGCAATAAAAAAAGGTCTTGAAA
TAGGTGAAATAGCCTGGGGAAAGATATTATTAGTTGAGGTTGAAAAGGGAGCTAAATTGCATCCATACATAGAATTATGGATT
TCGATAAAAATATAACAATATCCTCTTGAAAGTAAAGCTACTTTATGCTCTATACTGAAGGTAACAGGAAAATAAAAAAATAC
AAATGTGAATTCAGAAAGGAGAAAGAAAATGAAGGATAAGTATAAGGTACTTTATGACCCAATTAAAATTGGAAAATTGGAGA
TTAAAAATAGATATGTTCTTGCTCCAATGGGACCAGGAGGAATGTGTAACGCCGATGGCAGTTTTAATAAAAGAGGAATTGAG
TTTTATGTAGAACGTGCAAAAGGCGGAACTGGATTAATTATGACAGGTGTAACAATGGTGGAAAACAATATTGAAAAATGTGC
CCTGCCATCCATGCCATGTCCAACAATTAACCCTCTAAACTTTATTACAACAGGTAATGAAATGACAGAAAGAGTTCATGCAT
ATGGATCAAAAATATTTTTACAATTATCAGCAGGCTTTGGTAGAGTAAGTATACCATCTATTGTAGGAAAAGTGGCAGTAGCA
CCTTCTAAAATTCCACATAGATTTTTACCAGGAGTAACTTGTCGTGAGTTAACTACAGAAGAAGTAAAAGAATATGTAAAAGC
CTTTGGTGAATCAGCAGAAATTGCAAAAAAAGCCGGATTTGATGGTGTAGAAATTCATGCAGTACATGAAGGATACTTATTAG
ATCAATTTGCTATTTCTTTCTTTAACCATCGTACTGATGAATATGGTGGATCATTAGAAAATCGTTTAAGATTTGCCTGTGAG
GTTGTACAAGAAATTAAGAAACGTTGTGGACAAGACTTCCCAGTTTCACTTAGATACAGTATAAAAAGCTTCATTAAGGATTG
GTGTAAGGGTGGCTTACCAGATGAAGAATTTGAAGAAAAAGGAAGAGATATTCCAGAAGGAATTGAAGCTGCTAAAATACTTG
TTGCAGCAGGATATGATGCTTTAAATGGAGATGTTGGGTCTTATGATTCATGGTATTGGAGTCATCCACCAATGTATCAAAAG
AAGGGATTATACCTTCCATACAATGAAATACTTAAAAAGGTAGTAGATGTACCTATTATTACAGCAGGAAGAATGGAAGACCC
TGAACTATCAAGTGATGCAATTTTGTCAGGAAAAACAGATATGATTGCTTTAGGAAGGCCACTTCTTGCAGATGCAGAAATTC
CAAATAAGATTTTTGAAGATAAATATGATAAAGTTAGACCTTGTTTATCTTGTCAAGAAGGGTGTATGGGAAGATTACAGAAT
TTTGCAACAGTATCCTGTGCAGTAAATCCTGCCTGTGGACGTGAAAAAGAGTATGGACTAAAAAAAGCAGAACAGATTAAGAA
AGTTCTTGTTGTAGGTGGCGGTGTTGCAGGAATGGAAGCTGCAAGAGTTACAGCTGTTCGTGGACACAAAGTAACATTGATTG
AAAAGAATGGTTATCTTGGTGGAAATATTGTACCAGGAGGAATTCCAGATTTCAAAGATGATGATCGTGCACTTGTTAAATGG
TATGAAGGAATATTGAAAGATTTAGGTGTTGAAATAAAATTAAATGTGGGTGCATCAAAGGAAAATATCAAAGAATTTGGAGC
GGATGAAGTGCTTTTAGCAACAGGCTCTAGTCCAAGAACATTGACTATTGAAGGAGCAGATAAGGTTTATTCAGCAGAAGATG
TGTTAATGGAAAGAAAAACTGTTGGTGAAAAAGTTATTGTGATTGGTGGAGGACTTGTTGGATGTGAAACAGCTCTTTGGTTA
AAACAACAAGGTAAAGAGATTACAATTGTAGAGATGCAAAATGATATCCTGCAAGTAGGTGGACCTTTATGTCATGCAAACCA
CGATATGCTTGTTGATTTAATTAAATTCAATAAGATTGATGTTAAGACAAGCTCCTATATCAGCAAGAAAACAGATGAAGGAT
TTGTTTTAAATACAAATGGAGAAGAATCAATTATTAATGCTGATAGTGCTGTTGTAGCTATTGGATATTTATCTGAAAAAGAC
TTATATAGTGAAGTTAGATTTGATATTCCAAATGCAAGACTAATTGGAGATGCTAATAAAGTTCAAAATATTATGTATGCTAT
TTGGAGTGCATATGAAGTAGCTAAAAATATTTAAATAAACTTTTGCAAAAAACTAGAATGGGTTTATATACATATCTAGTTTT
TTGTTTTTTACTGAAATTCTATAAGTATATGAGTGTTTGTTATATATTTATTATAAAATACTAATTATTTAAAAATTTATTTA
ATGAATCATTATCTTTAAATAGGTTTCTTAAGTTCCCATATTTTTGTTCTAAATTTTTTCTATCATATTTATAGGATTTTTCT
CCTATATTCTCTACATTAAATTTAATAATTTCAGCATTTGATATAAGATATAATAATACCACTGCATTCTTTTCTAAAAAATT
GTTAGCTTTTTTATCATTCCAAAATTTATTATAATTTTCTTCTCCTAAATTTTCATTTGTTTTATAGTTAACGGTTATTTCAT
AAGGTTCTTTAGTAGTTTGTAGACTAAATCCAGCACT
```

Primary amino acid sequence of *Cs*fldZ

```
MKDKYKVLYDPIKIGKLEIKNRYVLAPMGPGGMCNADGSFNKRGIEFYVERAKGGTGLIMTGVTMVENNIEKCALPSMPCPTI
NPLNFITTGNEMTERVHAYGSKIFLQLSAGFGRVSIPSIVGKVAVAPSKIPHRFLPGVTCRELTTEEVKEYVKAFGESAEIAK
KAGFDGVEIHAVHEGYLLDQFAISFFNHRTDEYGGSLENRLRFACEVVQEIKKRCGQDFPVSLRYSIKSFIKDWCKGGLPDEE
FEEKGRDIPEGIEAAKILVAAGYDALNGDVGSYDSWYWSHPPMYQKKGLYLPYNEILKKVVDVPIITAGRMEDPELSSDAILS
GKTDMIALGRPLLADAEIPNKIFEDKYDKVRPCLSCQEGCMGRLQNFATVSCAVNPACGREKEYGLKKAEQIKKVLVVGGGVA
GMEAARVTAVRGHKVTLIEKNGYLGGNIVPGGIPDFKDDDRALVKWYEGILKDLGVEIKLNVGASKENIKEFGADEVLLATGS
SPRTLTIEGADKVYSAEDVLMERKTVGEKVIVIGGGLVGCETALWLKQQGKEITIVEMQNDILQVGGPLCHANHDMLVDLIKF
NKIDVKTSSYISKKTDEGFVLNTNGEESIINADSAVVAIGYLSEKDLYSEVRFDIPNARLIGDANKVQNIMYAIWSAYEVAKN
I
```

2. Sequence comparison between clostridial 2-enoate reductases and OYEs

```
C. sporogenes        ---------MKDKYKVLYDPIKIGKLEIKNRYVLAPMGPGGMCNADGSFNK-RGIEFYVE 50
C. tyrobutyricum     -----------MKNKSLFEPIKIGKVEVKNKISMAPMGAFGLVDNEGCYNQ-RAVDYYVE 48
C. kluyveri          -----------MKNKSLFEPIKIGNMEVKNKIAMAPMGAFGLVDNECCFNQ-RAVDYYVE 48
M. thermoacetica     ----------MVAYTRLFEPIKIGKVEIKNKIAMTPMGVLGLATHDGCFSK-RVVDYYVE 49
PETNR                ------------SAEKLFTPLKVGAVTAPNRVFMAPLTRLRSIEPGDIPTP-LMGEYYRQ 47
OYE2                 MSFVKDFKPQALGDTNLFKPIKIGNNELLHRAVIPPLTRMRAQHPGNIPNRDWAVEYYAQ 60

C. sporogenes        RAKGGTGLIMTGVTMVENNIEKCALPSMPCPTINPLNFITTGNEMTERVHAYGSKIFLQL 110
C. tyrobutyricum     RAKGGTGLIITSITKVENEIDKVVPGVIPIISINPGRFIMTSSEMTERVHAYGSKIFLQL 108
C. kluyveri          RAKGGTGLIITSVVKVENELDKVLTGVLPITSINPAKFIMTSSEMTERVHAYGSKIFLQL 108
M. thermoacetica     RAKGGTGLIITSVTKVDNEIERFKAGAVPVATANPLHFIATAGELTERVHAYGTKIFLQL 109
PETNR                RAS--AGLIISEATQISAQAKGYAGAPGLHSPEQIAAWKKITAGVHAEDGRIAVQLWHTG 105
OYE2                 RAQRPGTLIITEGTFPSPQSGGYDNAPGIWSEEQIKEWTKIFKAIHEKKSFAWVQLWVLG 120

C. sporogenes        SAGFGRVSIP-SIVGKV---------AVAPSKIPHRFLPGVTCRELTTEEVKEYVKAFGE 160
C. tyrobutyricum     TMGFGRSGAPGTLLTSQ---------PVSASSVPNYWDPTVTCRELTTSEVEWIVAKFIQ 159
C. kluyveri          SMGFGRSGAPGGLLTSQ---------PVSASAVPNYWDPTVTCRELTTSEVEWIVEKFAE 159
M. thermoacetica     GMGFGRVAAP-ILLESQ---------PVAPSALPNFWDPSITCRELTTAEVETLVQRASE 159
PETNR                RISHSSIQPGGQAPVSASALNANTRTSLRDENGNAIRVDTTTPRALELDEIPGIVNDFRQ 165
OYE2                 WAAFPDTLARDGLRYDS----ASDNVYMNAEQEEKAKKANNPQHSITKDEIKQYVKEYVQ 176

C. sporogenes        SAEIAKKAGFDGVEIHAVHEGYLLDQFAISFFNHRTDEYGGSLENRLRFACEVVQEIKKR 220
C. tyrobutyricum     GAAIAQKAGFDGVEIHAVHEGYLLDQFTLSIFNRRTDKYGGDLRGRLQLPIEIVQGIKAQ 219
C. kluyveri          GAKIAHKAGFDGVEIHAVHEGYLLDQFTLSIFNRRTDKYGGDLRGRLQLPIEIVEAIKTE 219
M. thermoacetica     AAEIAVEAGFDGVEIHAMHEGYLLDQFTIALFNRRGDKYGGALEDRLTFPIEIVRAIKDR 219
PETNR                AVANAREAGFDLVELHSAHG-YLLHQFLSPSSNQRTDQYGGSVENRARLVLEVVDAVCNE 224
OYE2                 AAKNSIAAGADGVEIHSANG-YLLNQFLDPHSNNRTDEYGGSIENRARFTLEVVDAVVDA 235

C. sporogenes        CGQDFPVSLRYSIKSFIKDWCKGGLPDEEFEEKGRDIPEGIEAAKILVAAGYDALNGDVG 280
C. tyrobutyricum     VGSDFPVGLRYSVKSCIKDWRQGGLPDEDYVEKGRDLEEGLESPQILEAAGYDELNTDVG 279
C. kluyveri          VGSNFPVGLRYSVKSCIKDWGQGGLAEEDYVEKGRDLEEGLEAAKILEAAGYDAFNADLG 279
M. thermoacetica     VGKDFPVVLRFSIKNYIKDWRQGGLPGENFQEKGRDVEEPLAAAKILEGAGYDGFDADAG 279
PETNR                WSADR-IGIRVSPIGTFQNVD-NGPNE--EADALYLIEELAKRG-----IAYLHMSETDL 275
OYE2                 IGPEK-VGLRLSPYGVFNSMS-GGAETGIVAQYAYVLGELERRAKAGKRLAFVHLVEPRV 293

C. sporogenes        SYDSWYWSHPPMYQKKGLYLPYNEILKKVVDVPIITAG-RMEDPELSSDAILSGKTDMIA 339
C. tyrobutyricum     TYDAWYWSHPPLYQKDGLYLPYTQELEKVVKIPVIVAG-KLGVPQEAEKALDEGGADMIG 338
C. kluyveri          TYDAWYWAHPPLYQKDGLYLPYTKELKKVVKIPVMVAG-KMGMPDVAEGALEDEAADMVT 338
M. thermoacetica     SYDAWYWAHPPVYQKHGCYLPLTQRLKEVVKVPVIVAG-RLEIPELAEEALVKGQADMIA 338
PETNR                A------GGKPYSE------AFRQKVRERFHGVIIGAG--AYTAEKAEDLIGKGLIDAVA 321
OYE2                 TNPFLTEGEGEYNG------GSNEFAYSIWKGPIIRAGNFALHPEVVREEV-KDPRTLIG 346

C. sporogenes        LGRPLLADAEIPNKIFEDKYDKVRPCLSCQEGCMGRLQNFATVSCAVNPACGREKEYGLK 399
C. tyrobutyricum     LARPLLSDAYWPKKVLSGHPERIRPCIGCHVACLGRGFEGKPLSCAVNPAAGRERYYEIR 398
C. kluyveri          LGRPLLCDAYWPKKVFTGQIDRIRPCIGCHTGCMGRGFEGRPLSCTVNPAAGRERYYEVK 398
M. thermoacetica     IGRGLLTDPYWVNKVMTGRSKNIRPCIGCHDGCLGRGFLGRPLSCTVNPACGREEEYAID 398
PETNR                FGRDYIANPDLVARLQKKAELNPQRPESFYGGGAEGYTDYPSL----------------- 364
OYE2                 YGRFFISNPDLVDRLEKGLPLNKYDRDTFYKMSAEGYIDYPTYEEALKLGWDKH------ 400

C. sporogenes        KAEQIKKVLVVGGGVAGMEAARVTAVRGHKVTLIEKNGYLGGNIVPGGIPDFKDDDRALV 459
C. tyrobutyricum     PAAIPKKVLIAGGGVAGMEAARMAVLRGHKVTLYESTDQLGGEIVPGSVPDFKIDDRRLL 458
C. kluyveri          PAAAPKKVMIVGGGVAGMEAARITAMRGHKVSMYEGTKELGGQVIPASVPDFKIDDRRLL 458
M. thermoacetica     RAPEAKQVMVIGGGVAGMEAARVPALRGHRVSLYEKSDRLGGHVVEAAVPDFKADDGRLL 458
PETNR                ------------------------------------------------------------
OYE2                 ------------------------------------------------------------

C. sporogenes        KWYEGILKDLGVEIKLNVGASKENIKEFGADEVLLATGSSPRTLTIEGA--DKVYSAEDV 517
C. tyrobutyricum     DWYRNEMKELKINVIFNTEVTDKLVGKEQPDVVIVATGANDVKIKLPGMEKDKVSTAVDI 518
C. kluyveri          DWYRNEMKELKVKLVLDTNVTEEVVEKEKPDVVIIATGAKEIKLNLPGIEKDKVATVIEV 518
M. thermoacetica     EWYKTELGELQVEIHLNQEVTPEFVEEKNPDVVVVATGSTPAIPDIPGVNKDKVTTVSDL 518
PETNR                ------------------------------------------------------------
OYE2                 ------------------------------------------------------------

C. sporogenes        LMERKTVGEKVIVIGGGLVGCETALWLKQQGKEITIVEMQNDILQVGGPLCHANHDMLVD 577
C. tyrobutyricum     LNGTKKSGKNVLIVGGGLVGCETALYLAKAGKKVAIVEAKDKILDAGKPIPHMNKIMLED 578
C. kluyveri          LKGSKQVGENVLMVGGGLAGCETALYLAKQGKKVTIIEARDTILNAGKPVPHMNKIMLID 578
M. thermoacetica     LLGKKQAGDRVVIIGGGLVGCETALWLAQQGKDVTIIEILDDLMRAGIPVPYMNRMMLLD 578
PETNR                ------------------------------------------------------------
OYE2                 ------------------------------------------------------------

C. sporogenes        LIKFNKIDVKTSSYISKKTDEGFVLN-TNGEESIINADSAVVAIGYLSEKDLYSEVRFDI 636
C. tyrobutyricum     LIKKYNIKVITGNSLLEVTDAGAVLIDSKFKQQEVSADTVVISIGFKSNRKLYNKLHGKV 638
C. kluyveri          LLKNSGVNIITETSLLEVTDRGAILIDNKFKKQNIDADTVVIAVGFKADRELYNKLRDKV 638
M. thermoacetica     LLKMNGVKWLTETSVLEVTDDGVTLIGKNYQRSPLPADTVILAVGFGADQRLYNALRDKI 638
PETNR                ------------------------------------------------------------
OYE2                 ------------------------------------------------------------

C. sporogenes        PNARLIGDANKVQNIMYAIWSAYEVAKNI 665
C. tyrobutyricum     TDLYLIGDAYQAANIMDAIWSGNEIGLNC 667
C. kluyveri          ADLYLVGDANESANIMNAIWSANEIALNC 667
M. thermoacetica     PNLYLIGDSREPRNILAGIWEGYEVGRGI 667
PETNR                -----------------------------
OYE2                 -----------------------------
```

Fig. S1 Alignment showing homology between clostridial 2-enoate reductases and OYE-reductases. Functional residues are highlighted: GREEN - 4Fe-4S cluster binding site; BLUE - FMN binding site; YELLOW - substrate binding and active site; GRAY - FAD binding domain. Analysed by Conserved Domain Search Service (http://www.ncbi.nlm.nih.gov/Structure/cdd/wrpsb.cgi).

3. Overexpression of *Cs*fldZ

E. coli-codon optimised *Cs*fldZ

ATGAAAGACAAATACAAAGTGCTGTATGACCCGATTAAAATCGGCAAATTGGAAATCAAGAATCGCTATGTCCTGGCACCTATGGGGCCAGGCG
GGATGTGCAATGCAGATGGGTCCTTTAACAAACGTGGGATCGAATTCTATGTTGAACGCGCAAAAGGCGGTACTGGCCTGATTATGACGGGTGT
CACGATGGTGGAAAACAACATTGAGAAATGTGCGCTCCCTAGCATGCCCTGTCCCACTATCAACCCGCTGAATTTCATTACCACCGGGAACGAA
ATGACCGAACGTGTGCACGCGTACGGTTCGAAAATTTTCCTGCAACTTTCAGCGGGCTTTGGCCGCGTATCCATTCCAAGTATCGTTGGGAAAG
TAGCGGTGGCACCCTCGAAGATCCCGCATCGCTTTCTGCCAGGTGTCACCTGCCGTGAACTGACCACCGAAGAAGTGAAAGAGTACGTTAAGGC
GTTTGGTGAGTCTGCCGAAATCGCGAAGAAAGCGGGTTTTGATGGAGTGGAAATTCATGCCGTCCACGAAGGCTATCTGCTGGATCAGTTTGCT
ATCTCTTTCTTTAACCACCGTACCGATGAATATGGCGGTTCTCTGGAAAACCGCTTACGTTTTGCGTGCGAGGTGGTTCAAGAGATTAAAAAGC
GCTGTGGTCAGGACTTCCCGGTTTCTCTGCGCTACAGCATTAAGAGCTTTATCAAGGACTGGTGCAAAGGAGGCTTACCGGACGAAGAATTCGA
AGAGAAAGGCCGGGATATTCCAGAAGGTATTGAGGCCGCCAAAATTCTGGTGGCTGCGGGGTATGATGCCCTGAACGGCGACGTGGGCAGTTAT
GATAGCTGGTATTGGTCGCATCCTCCGATGTACCAGAAAAAAGGGCTCTACTTACCGTATAACGAGATTCTGAAAAAGGTGGTGGATGTCCCGA
TTATCACCGCAGGCCGTATGGAGGATCCAGAACTGAGCAGCGATGCCATTCTTTCTGGCAAAACGGACATGATTGCGCTTGGACGCCCGCTCTT
GGCTGATGCGGAGATTCCGAATAAAATCTTTGAAGATAAGTACGACAAAGTCCGCCCGTGCCTTTCATGCCAGGAAGGCTGTATGGGTCGTCTG
CAGAACTTTGCTACCGTTAGTTGTGCAGTAAATCCTGCATGCGGTCGCGAAAAAGAATACGGTTTAAAGAAAGCGGAACAGATCAAAAAAGTAC
TGGTGGTTGGCGGCGGTGTAGCGGGTATGGAAGCTGCCCGCGTCACAGCCGTTCGTGGCCACAAAGTGACCCTGATTGAAAAGAACGGCTATTT
AGGCGGGAATATCGTACCGGGAGGCATTCCGGACTTTAAAGACGATGATCGTGCACTGGTTAAATGGTATGAAGGCATTCTGAAAGATCTGGGC
GTCGAAATCAAACTGAATGTGGGTGCGAGCAAAGAGAACATCAAAGAGTTCGGGGCTGACGAAGTGCTCTTAGCTACAGGAAGCAGTCCGCGCA
CATTGACGATTGAGGGTGCGGACAAAGTTTATTCGGCCGAAGATGTGTTAATGGAACGGAAAACGGTCGGTGAGAAAGTGATCGTTATTGGTGG
CGGCTTGGTTGGTTGCGAAACTGCACTTTGGCTGAAACAACAGGGCAAAGAAATCACCATTGTGGAAATGCAAAACGATATCCTGCAAGTGGGT
GGACCGCTCTGTCATGCAAACCATGACATGTTGGTTGACTTGATTAAATTTAACAAAATTGATGTCAAAACTTCCTCGTACATTTCCAAAAAAA
CGGATGAAGGCTTCGTCTTGAATACGAATGGTGAGGAAAGCATTATCAATGCCGATAGTGCTGTGGTTGCCATCGGATACCTGTCAGAGAAAGA
TCTGTACTCCGAGGTACGCTTCGATATCCCCAATGCCCGTCTGATTGGTGATGCGAATAAGGTCCAGAACATCATGTATGCGATTTGGTCAGCC
TATGAAGTAGCGAAGAATATTTGA

Affinity tags

DNA sequences encoding affinity tags flanked by *Xba*I and *Nde*I restriction sites were custom synthesised as gBlocks (IDT DNA), digested with restriction enzymes and ligated upstream of *Cs*fldZ coding sequence in pET20b(+).

>S-tag
CAATTCCCCTCTAGAAATAATTTTGTTTAACTTTAAGAAGGAGATATACCATGAAAGAAACCGCTGCTGCTAAATTCGAACGC
CAGCACATGGACAGCCCAGATCTGGGTACCCTGGTGCCACGCGGTTCCCATATGGCTAGCATGACT

>GST-tag
GATCTCGTCTAGAAATAATTTTGTTTAACTTTAAGAAGGAGATATACCATGTCCCCTATACTAGGTTATTGGAAAATTAAGGG
CCTTGTGCAACCCACTCGACTTCTTTTGGAATATCTTGAAGAAAAATATGAAGAGCATTTGTATGAGCGCGATGAAGGTGATA
AATGGCGAAACAAAAAGTTTGAATTGGGTTTGGAGTTTCCCAATCTTCCTTATTATATTGATGGTGATGTTAAATTAACACAG
TCTATGGCCATCATACGTTATATAGCTGACAAGCACAACATGTTGGGTGGTTGTCCAAAAGAGCGTGCAGAGATTTCAATGCT
TGAAGGAGCGGTTTTGGATATTAGATACGGTGTTTCGAGAATTGCATATAGTAAAGACTTTGAAACTCTCAAAGTTGATTTTC
TTAGCAAGCTACCTGAAATGCTGAAAATGTTCGAAGATCGTTTATGTCATAAAACATATTTAAATGGTGATCATGTAACCCAT
CCTGACTTCATGTTGTATGACGCTCTTGATGTTGTTTTATACATGGACCCAATGTGCCTGGATGCGTTCCCAAAATTAGTTTG
TTTTAAAAAACGTATTGAAGCTATCCCACAAATTGATAAGTACTTGAAATCCAGCAAGTATATAGCATGGCCTTTGCAGGGCT
GGCAAGCCACGTTTGGTGGTGGCGACCATCCTCCAAAATCGGATCTTGAAGTCCTCTTTCAGGGACCCCTGCATATGGCTAGC
A

>Strep-tag
GAGCGGATAACAATTCCCCTCTAGAAATAATTTTGTTTAACTTTAAGAAGGAGATATACCATGGCAAGCTGGAGCCACCCGCA
GTTCGAAAAGGGTGCACTTGAAGTCCTCTTTCAGGGACCCGGGCATATGGCTAGCATGACTGGTGGA

>T7-tag
GAGCGGATAACAATTCCCCTCTAGAAATAATTTTGTTTAACTTTAAGAAGGAGATATACCATGGCTAGCATGACTGGTGGACA
GCAAATGGGTCGCGGACATATGTCCGGCTGCTAACAAAGCCCGAAAGGAA

>NtH-tag
GAGCGGATAACAATTCCCCTCTAGAAATAATTTTGTTTAACTTTAAGAAGGAGATATACCATGGGCAGCAGCCATCATCATCA
TCATCACAGCAGCGGCCTGGTGCCGCGCGGCAGCCATATGAAA

C-terminally 6xHis-tagged *Cs*fldZ expression construct was created by PCR reaction with primers PM017 (5'-aaacatATGAAAGACAAATACAAAGTGCTGTATGAC-3') and PM019 (5'-AAAGCGGCCGCTCAAATATTCTTCGCT ACTTCATAGGCTGAC-3'), digestion of the product with *Nde*I and *Not*I and ligation to pET20b(+) linearized with the same enzymes.

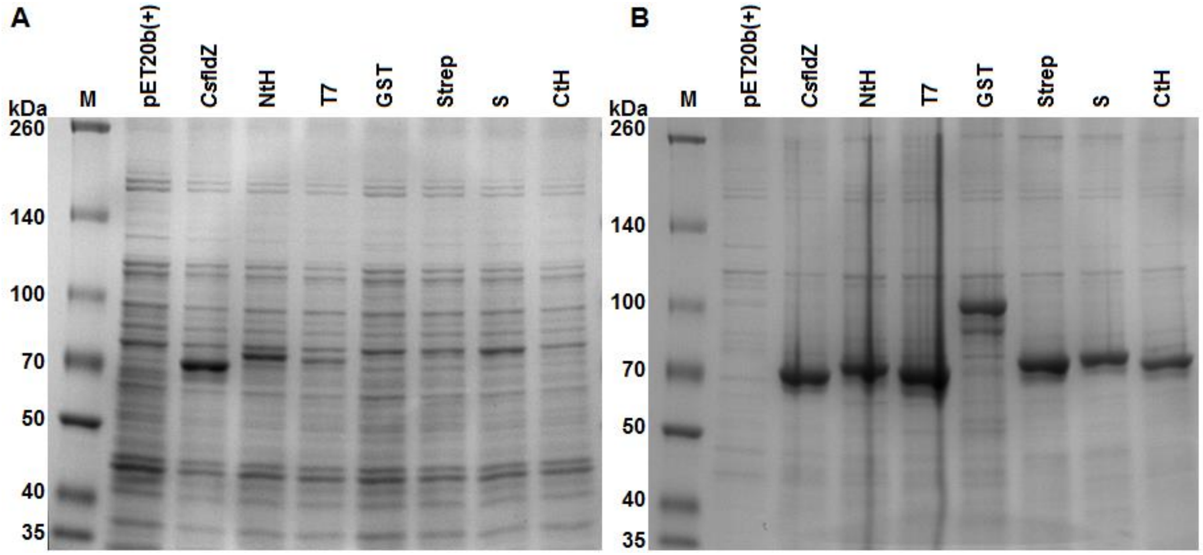

Fig. S2 Overexpression of *Cs*fldZ with different affinity tags. **A** – soluble protein fraction, **B** – insoluble protein fraction. Lines and expected protein bands: Protein marker Spectra™ Multicolor Broad Range Protein Ladder; pET20b(+) empty plasmid control; untagged *Cs*fldZ (73 kDa); N-terminal His-tag (75.2 kDa); N-terminal T7-tag (74.5 kDa); N-terminal GST-tag (99.8 kDa); N-terminal Strep-tag (75.6 kDa); N-terminal S-tag (76.1 kDa) and C-terminal His-tag (74.3 kDa).
